# Viewpoint-Agnostic Taekwondo Action Recognition Using Synthesized Two-Dimensional Skeletal Datasets

**DOI:** 10.3390/s23198049

**Published:** 2023-09-23

**Authors:** Chenglong Luo, Sung-Woo Kim, Hun-Young Park, Kiwon Lim, Hoeryong Jung

**Affiliations:** 1 Division of Mechanical and Aerospace Engineering, Konkuk University, 120 Neungdong-ro, Gwangjin-gu, Seoul 05029, Republic of Korea; luo0611@konkuk.ac.kr; 2 Physical Activity and Performance Institute, Konkuk University, 120 Neungdong-ro, Gwangjin-gu, Seoul 05029, Republic of Korea; kswrha@konkuk.ac.kr (S.-W.K.); parkhy1980@konkuk.ac.kr (H.-Y.P.); exercise@konkuk.ac.kr (K.L.); 3 Department of Sports Medicine and Science, Graduate School, Konkuk University, 120 Neungdong-ro, Gwangjin-gu, Seoul 05029, Republic of Korea; 4 Department of Physical Education, Konkuk University, 120 Neungdong-ro, Gwangjin-gu, Seoul 05029, Republic of Korea

**Keywords:** Taekwondo poomsae, action recognition, skeletal data, camera viewpoint, martial arts

## Abstract

Issues of fairness and consistency in Taekwondo poomsae evaluation have often occurred due to the lack of an objective evaluation method. This study proposes a three-dimensional (3D) convolutional neural network–based action recognition model for an objective evaluation of Taekwondo poomsae. The model exhibits robust recognition performance regardless of variations in the viewpoints by reducing the discrepancy between the training and test images. It uses 3D skeletons of poomsae unit actions collected using a full-body motion-capture suit to generate synthesized two-dimensional (2D) skeletons from desired viewpoints. The 2D skeletons obtained from diverse viewpoints form the training dataset, on which the model is trained to ensure consistent recognition performance regardless of the viewpoint. The performance of the model was evaluated against various test datasets, including projected 2D skeletons and RGB images captured from diverse viewpoints. Comparison of the performance of the proposed model with those of previously reported action recognition models demonstrated the superiority of the proposed model, underscoring its effectiveness in recognizing and classifying Taekwondo poomsae actions.

## 1. Introduction

Taekwondo is a traditional Korean martial art that has become one of the most popular sports worldwide. Two types of Taekwondo competitions are conducted: gyeorugi and poomsae, which involve various movements and complex techniques. Gyeorugi requires two competing players, and objective judgments are made using a quantitative and accurate electronic scoring system. In poomsae, a single player demonstrates basic attack and defense techniques in a specific order. In this case, evaluation is subjective and qualitative, based on the opinions of the judges, except for penalties (e.g., stopping or crossing the boundaries). Owing to situational constraints, judges must evaluate multiple participants simultaneously, which may give rise to concerns of unfairness and inconsistencies in evaluations not only in competitions but also in promotional tests. To address these issues, quantitative evaluation methods using vision-based action recognition techniques have been proposed [1,2].

Vision-based human action recognition (HAR) has emerged as a prominent area of interest in computer vision and artificial intelligence. Its primary objective is to detect and analyze human actions from unknown video sequences, thereby enabling a deeper understanding and interpretation of such actions. HAR has been applied in various domains, including security [3,4,5], healthcare [6,7,8], and sports [9,10,11]. Vision-based HAR systems have been employed to support quantitative evaluation and judgment in various sports [12,13,14,15,16,17,18]. However, few studies have reported the application of action recognition technology in martial arts disciplines, such as Taekwondo [1,2]. In previous studies, action recognition approaches using RGB (color) and RGB-D (color and depth) images have been proposed. These included methods that emphasized the dominant poses associated with each action in RGB-D videos as input to a convolutional neural network (CNN) [19], as well as techniques that enhanced the structural information of body parts, joints, and temporal scales by representing sequences of depth maps as structured dynamic images [20]. However, the rapid-action characteristics of martial arts pose challenges to motion capture because of insufficient sharpness and intermittent frame loss. The dynamic nature of martial arts and wide range of actions possible therein render the RGB-D methods inadequate. Furthermore, these approaches are susceptible to domain shifts caused by environmental changes and cannot accurately predict dynamic actions.

Recent research on action recognition has incorporated skeletal data into complex human action recognition [21,22,23,24,25,26,27,28,29,30]. For instance, Du et al. proposed an architecture that divided the human skeleton into five parts and fed them into separate subnetworks instead of using recurrent neural networks to process the entire skeleton as input [28]. Yan et al. introduced an action recognition method based on graph convolutional networks (GCNs) considering the spatiotemporal features of skeletons [19]. Subsequently, GCN-related studies [23,25,26,27,29], including one by Duan et al., generated heatmaps using skeletons to address the limitations of the GCN methods, such as the accuracy of skeleton coordinates and integration with other modality data [22]. In skeleton-based action recognition, the skeleton representation provides core information that is highly relevant to human behavior. Unlike the RGB-D models, it remains robust against variations in illumination, changes in clothing, and environmental factors.

Previous approaches used for action recognition primarily relied on images obtained from a single viewpoint. However, in the context of poomsae evaluation, the same movement may appear different when captured from different viewpoints, thereby posing challenges for accurate recognition. Furthermore, single-view action recognition requires training models specific to each viewpoint, thereby necessitating retraining efforts while dealing with images captured from other viewpoints. This results in potential time and resource constraints. Moreover, single-view action recognition predominantly focuses on discerning individual movements and presents difficulties in recognizing complex movements involving multiple actions.

In this study, we propose a novel action recognition model for the evaluation of poomsae that exhibits robust recognition performance regardless of the variations in viewpoints. The model uses three-dimensional (3D) skeletons collected using a full-body motion-capture suit to create two-dimensional (2D) skeletons from a desired viewpoint. Thus, the proposed approach obtains 2D skeletal data from diverse viewpoints as part of the training data and effectively addresses the effect of observational viewpoints, ensuring consistent and reliable performance in action recognition regardless of the viewpoint. The main contributions of this study are as follows:

1. A 3D skeletal dataset comprising 16 unit actions in Taekwondo poomsae was constructed using motion data collected by employing full-body motion-capture suits.
2. Methods were proposed for generating 2D skeletons by projecting 3D skeletons from diverse viewpoints, which in turn were used to generate synthetic joint and bone heatmaps. These incorporated viewpoint-dependent action characteristics into the training dataset. This ensured consistent and reliable performance, regardless of the viewpoint.
3. The optimal camera viewpoint for action recognition of Taekwondo poomsae was determined by analyzing and evaluating the recognition performance.

## 2. Materials and Methods

### 2.1. Data Collection

**Figure 1:**
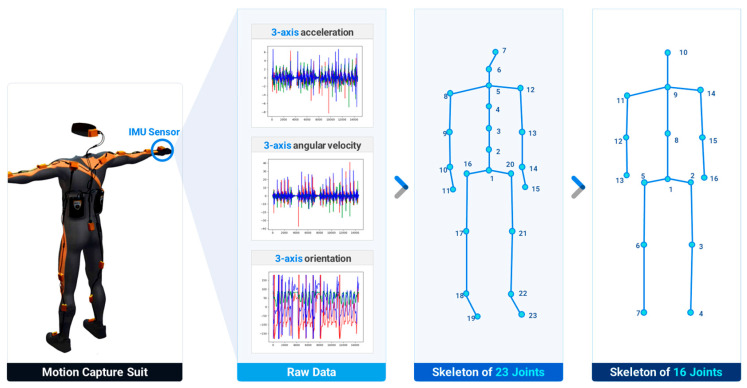
3D skeleton data-collection procedure.

**Figure 2:**
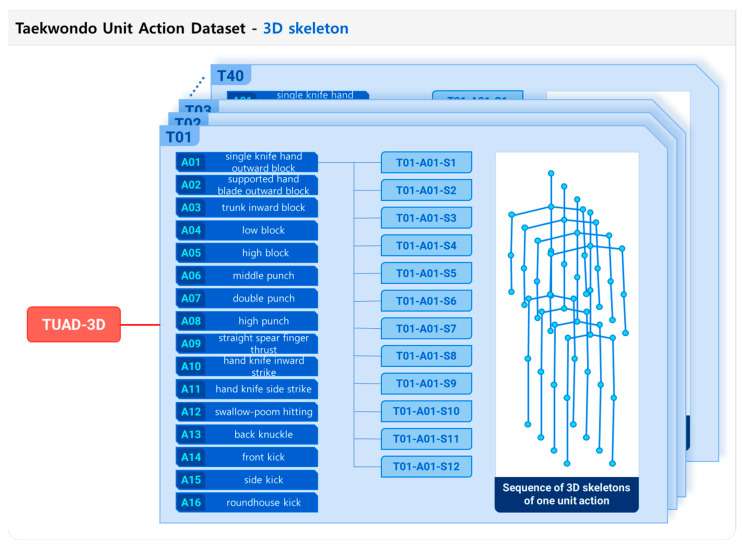
Structure of Taekwondo unit-action dataset of 3D skeletons (TUAD-3D).

Primary motion data were collected from Taekwondo experts using a full-body motion-capture suit (Xsens MVN; Xsens Corp., Enschede, The Netherlands). The suit was equipped with 17 inertial measurement unit (IMU) sensors, where each sensor measured the acceleration, angular velocity, and orientation of the body segment at the attached point along three mutually perpendicular axes. Subsequently, the raw data obtained from the motion-capture suit were processed to extract the positions of 23 joints in the human skeleton [31]. To enhance the generalizability of the action recognition model, the skeleton with 23 joints was converted to one with 16 joints, as illustrated in Figure 1. Forty Taekwondo experts participated in the data collection. The data-collection procedure involved in this research was approved by the Konkuk University Institutional Review Board (IRB) under protocol number 7001355-202004-HR-372. The 3D skeleton data gathered using the motion-capture suit did not contain any personal privacy information. Furthermore, informed consent was obtained from each participant prior to the data collection, and we made a commitment to use the data exclusively for academic research purposes. Each subject was instructed to sequentially perform the 16 unit actions of Taekwondo poomsae as a predefined data-collection protocol while wearing the motion-capture suit. Each subject executed each action 12 times and repeated the process twice, thereby generating 12 sets of executions for each unit action. Consequently, a Taekwondo unit-action dataset comprising 7680 unit-action data points (16 unit actions per subject × 12 repetitions × 40 participants) was prepared; finally, a motion database, named the Taekwondo unit action dataset of 3D skeletons (TUAD-3D), was constructed, as depicted in Figure 2.

#### 2.1.1. 3D CNN-Based Viewpoint-Agnostic Action Recognition

**Figure 3:**
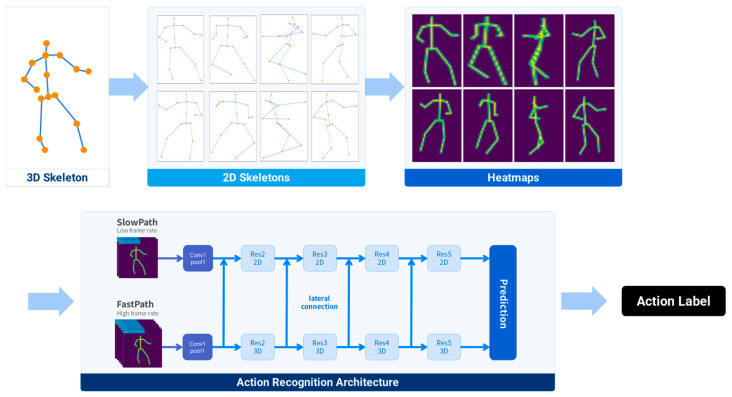
Overall architecture of the 3D CNN-based viewpoint-agnostic action recognition.

The viewpoint-agnostic action recognition proposed in this study adopted a previously reported posec3d framework, which utilized a sequence of 2D skeleton heatmaps as input to a 3D CNN as the primary action recognition architecture [22]. To address the performance degradation caused by viewpoint mismatch in training and test images, this study proposed a method for using diverse-viewpoint 2D skeletons generated through the projection of 3D skeletons as the training dataset. Finally, the 2D skeletons were converted into synthetic heatmap images and used to train the action recognition network. Figure 3 illustrates the action recognition architecture proposed in this study.

#### 2.1.2. Generation of Diverse-Viewpoint 2D Skeletons from 3D Skeleton

**Figure 4:**
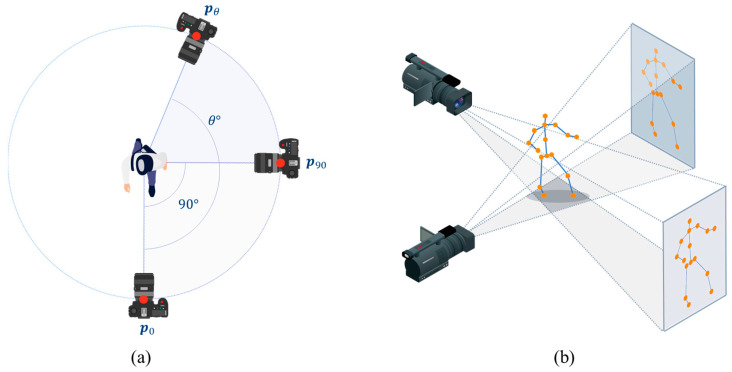
Generation of 2D skeletons by projecting a 3D skeleton onto various image planes: (**a**) determination of camera viewpoints by rotating the initial viewpoint; (**b**) projection of the 3D skeleton onto the desired image planes.

Figure 4 illustrates the projection of the 3D skeleton onto the image planes of various camera viewpoints to generate 2D skeletons with diverse viewpoints. In this procedure, we assumed that the camera could be rotated along a fixed orbit around the center of the 3D skeleton, as depicted in Figure 4a. The position of the camera was calculated by multiplying the rotation matrix $R_{z}\left(\theta \right)$ with its initial position $p_{0}$, as follows:

(1) $$p_{\theta }=R_{z,\theta }\;p_{0},$$

where $p_{0}$ and $p_{\theta }$ denote the initial and rotated camera positions, respectively. To incorporate various perspectives, the joint positions of the 3D skeleton were rotated in intervals of 10°, 45°, and 90°. This rotation facilitated the projection of the 3D skeleton keypoints onto a 2D image plane, thereby transforming the 3D skeleton information into corresponding 2D skeleton information. Although the process of rotation resulted in a partial loss of position and orientation information of the rotated skeleton, it effectively enabled the representation of 2D skeleton information from diverse viewpoints. The 2D skeleton at the rotated camera position $p_{\theta }$ was projected by multiplying the projection matrix $P_{\theta }$ with the 3D skeleton coordinates:

(2) $$s_{i,\theta }^{2D}=P_{\theta }\;s_{i}^{3D},$$

where $s_{i}^{3D}$ denotes the *i*th joint position of the 3D skeleton, \ and $s_{i,\theta }^{2D}$ is the joint position of the 2D skeleton corresponding to the camera position $p_{\theta }$**.** The projection matrix $P_{\theta }$ can be acquired using intrinsic and extrinsic camera parameters. An intrinsic parameter characterizes the optical properties of the camera, while an extrinsic parameter matrix describes its position and orientation.

#### 2.1.3. Generation of Synthetic Heatmap Image from 2D Skeleton

The joint positions of the 2D skeletons were employed to generate synthetic 2D heatmap images. The value assigned to each pixel coordinate within the heatmap image was determined by applying a Gaussian kernel to that coordinate. The heatmaps generated were categorized into bone and joint heatmaps. To reduce the volume of the 3D heatmaps, we implemented two techniques. The first technique involved subject-centered cropping, which involved cropping all the frames based on a minimum bounding box that enclosed the subject in a 2D pose. The frames were cropped as the participant moved within a confined area. Subsequently, the cropped frames were resized to the desired target size. The second technique involved uniform sampling, which entailed selecting a subset of frames to capture the temporal dimension. This sampling approach ensured that the frames were uniformly distributed throughout the sequence, thereby effectively reducing the computational load.

The bone heatmap serves as a visual representation of skeletal connectivity and depicts the interconnections among different segments of the skeleton, thereby facilitating the comprehension of its structural arrangement and tracking of joint movements. Conversely, the joint heatmap focuses on representing the central point of each skeletal segment. This enables the precise localization of joint positions and provides a more detailed understanding of the skeletal shape, which is utilized for motion analysis. The training and validation procedures were conducted separately for the two types of heatmaps to ensure their individual accuracies. The pixel value of the joint heatmap $J_{i,j}$ was calculated as follows:

(3) $$J_{i,j}=\sum_{k=1}^{NbJoint}\exp\left(-\frac{D\left(i,j,u_{k}\right)}{2\sigma^{2}}\right),$$

where σ is the variance of the Gaussian map and $D\left(i,\;j,u_{k}\right)$ represents the Euclidean distance between pixels $\left(i,j\right)$ and the *k*th joint position of the 2D skeleton ($u_{k}$). The pixel value of the bone heatmap $B_{i,\;j}$ was calculated as follows:

(4) $$B_{i,j}=\sum_{k=1}^{NbBone}\exp\left(-\frac{D\left(i,j,b_{k}\right)}{2\sigma^{2}}\right),$$

where $D\left(i,j,b_{k}\right)$ denotes the shortest distance between pixel $\left(i,j\right)$ and the *k*th bone segment $b_{k}$, which is defined by the two joint positions of the 2D skeleton. After the above-mentioned process, 2D joint and bone heatmaps were generated for each 2D skeleton. This process resulted in a 3D heatmap with dimensions of T×H×W for each action sequence, where T is the number of frames in each action and H and W represent the height and width of the heatmap image, respectively.

#### 2.1.4. 3D CNN Architecture

**Figure 5:**
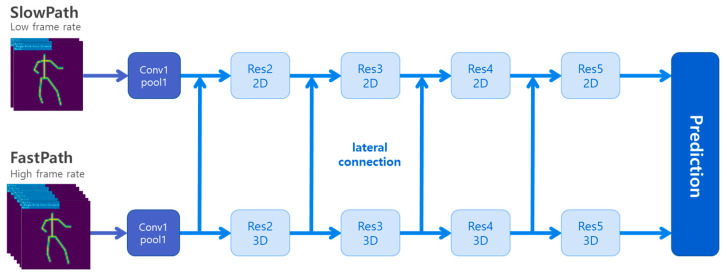
3D CNN SlowFast architecture.

The SlowFast architecture was employed to construct a 3D CNN action-classification model [32]. It comprises two distinct pathways, namely slow and fast, as illustrated in Figure 5. The slow pathway is designed to effectively retain spatial information, whereas the fast pathway preserves temporal information. By combining these two pathways, the SlowFast architecture possesses an enhanced capability in capturing both spatial and temporal features, resulting in improved accuracy for action-classification tasks.

#### 2.1.5. Training Procedure

**Figure 6:**
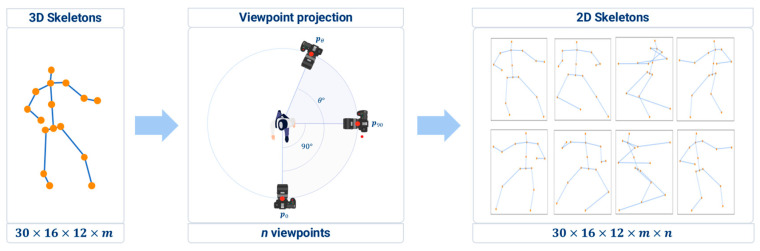
The number of skeletons used in the model training.

Three-dimensional skeletal databases of the 16 unit actions of poomsae, each performed 12 times and collected from 40 Taekwondo experts, were used to train the proposed action-classification model. Specifically, the action data of 30 experts were allocated for model training, while the remaining 10 experts were reserved for model testing. As illustrated in Figure 6, the 3D skeletal data were subjected to processing to derive a 2D skeleton representation, resulting in an expanded dataset size contingent on the number of projected viewpoints. The number of 2D skeletons used in the model training was 30×16×12×m×n, where *m* and *n* denote the number of frames in one action and the number of viewpoint projections, respectively. During the training phase, we assessed the generalization performance via a 5-fold cross-validation. The model was trained using the stochastic gradient descent optimizer with a maximum of 240 epochs, and cross-entropy loss was employed as the chosen loss function.

### 2.2. Evaluation Metrics

The evaluation metrics used in the experiment were *F1-score*, *precision*, *recall*, and *accuracy*, given as follows:

(5) $$precision=\frac{TP}{TP+FP},$$

(6) $$recall=\frac{TP}{TP+FN},$$

(7) $$accuracy=\frac{TP+TN}{TP+FP+TN+FN},$$

where *TP* (true positive) represents samples that are predicted as positive, and the ground truth also labels them as positive; FP (false positive) represents samples that are predicted as positive, but the ground truth labels them as negative; *TN* (true negative) represents samples that are predicted as negative, and the ground truth also labels them as negative; and *FN* (false negative) represents samples that are predicted as negative, but the ground truth labels them as positive. The *F1-score* is a metric that balances precision and recall and measures the performance of the model. A higher F1-score indicates better performance.

(8) $$F1-Score=\frac{2\times precision\times recall}{precision+recall}.$$

## 3. Results

The action recognition model was trained on four 2D skeletal datasets. Table 1 lists the configurations of the training datasets. Each training dataset comprised 2D skeletons generated by projecting a 3D skeleton at several viewing angles. The models trained using these four datasets were denoted as Models A–D, as listed in Table 1. The performances of these models were compared with one another to deduce the optimal configuration of the projection viewpoints of the 2D skeletons for the highest recognition performance.

### 3.1. Performance Evaluation Using Synthetic 2D Skeleton Datasets

The performance of the model was assessed using the synthesized 2D skeletal datasets mentioned earlier. The test samples for the 2D skeletal data were generated by projecting the 3D skeletons of ten individuals selected from TUHA-3D at 10° intervals across the viewpoints. Table 2 and Table 3 present the evaluation results of the joint and bone heatmaps, respectively. While training and testing with the joint heatmap, the highest performance was observed for Model D, with an accuracy of 0.9802. Similarly, while training and testing the bone heatmap, the highest performance was observed for Model D, with an accuracy of 0.9783. The performance comparison results show that the recognition accuracy increased as more 2D skeletons, projected at distinct viewing angles, were included in the training dataset.

### 3.2. Performance Evaluation Using 2D Skeletons Extracted from Front- and Side-View RGB Images

**Figure 7:**
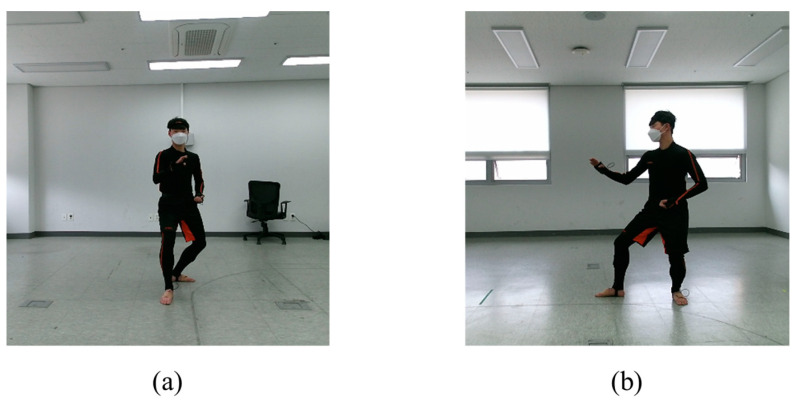
RGB image samples used for performance evaluation: (**a**) frontal and (**b**) lateral.

The performance of the proposed model was evaluated using 2D skeleton data extracted from RGB images. The test samples of the 2D skeleton data were extracted from the poomsae unit-action images captured using an RGB-D camera (Realsense 435d; Intel Corporation, Silicon Valley, CA, USA). In the data-collection procedure, two additional RGB-D cameras were installed, one in the front and one on the left-hand side of the participants, to collect test sample images. Figure 7 depicts the RGB images captured by the frontal and lateral cameras. Overall, 5527 test samples of 2D skeletons were generated from the RGB images using the HRnet pose-estimation algorithm. Table 4 and Table 5 present the evaluation results using the joint and bone heatmaps, respectively. While training and testing with the joint heatmap, the highest performance was observed for Model D, with an accuracy of 0.8705. Similarly, while training and testing the bone heatmap, the highest performance was observed for Model C, with an accuracy of 0.8761. The performance comparison results demonstrate the effectiveness of the action recognition model trained with synthetic 2D skeletons for the RGB test samples.

### 3.3. Performance Evaluation Using 2D Skeletons Extracted from Random-View RGB Images

**Figure 8:**
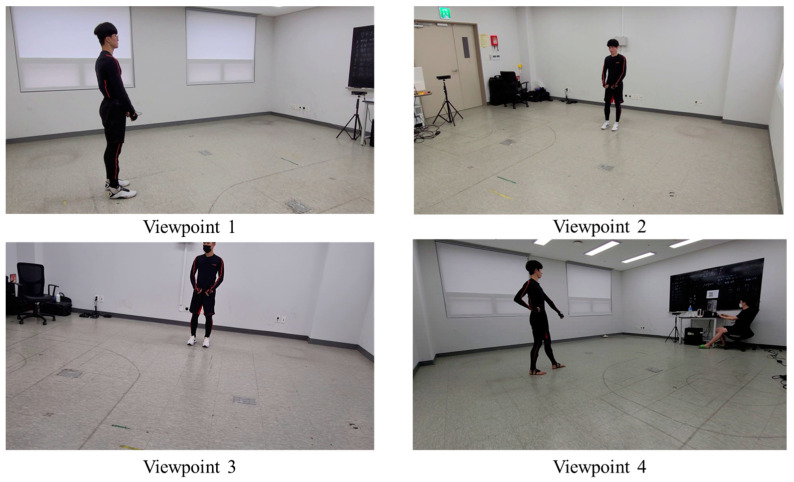
RGB image samples captured from random viewpoints for the performance evaluation of the model.

Next, the performance of the model was evaluated using a 2D skeletal dataset extracted from the RGB images captured from random viewpoints. The RGB image dataset, obtained using four smartphone cameras from four distinct viewpoints, was used for the assessment. The dataset comprised 639 samples of poomsae unit actions. Figure 8 illustrates the RGB image samples used for the performance evaluation. Table 6 and Table 7 present the evaluation results using the joint and bone heatmaps, respectively. While training and testing with the joint heatmap, the highest performance was observed for Model C, with an accuracy of 0.9381. Similarly, when training and testing the bone heatmap, the highest performance was observed for Model D, with an accuracy of 0.8670. A performance comparison shows that the action recognition model trained with synthetic 2D skeletons could work on the test samples obtained from random-view RGB images.

### 3.4. Performance Comparison with Previously Published Models

The performance of the proposed model was compared with those of previously reported action recognition models, including posec3d [22], stgcn [29], stgcn++ [33], ctrgcn [26], and aagcn [34]. To this end, the proposed model was trained using the 2D skeletal databases of Models C and D. The previous models were trained using the 2D skeletal databases extracted from the RGB images of the poomsae unit action captured from the frontal and lateral viewpoints presented in Section 3.2. The synthetic two-dimensional skeletal dataset and random-viewpoint RGB image datasets were used as test datasets for the proposed and previously reported models. The detailed outcomes of this evaluation for the synthetic two-dimensional skeletal test dataset can be found in Table 8. It is noteworthy that both Model C and Model D achieved accuracies surpassing 0.97 within the table. The specific results of this evaluation for the random-view RGB image test dataset are enumerated in Table 9. Notably, the proposed model trained with the 2D skeletal databases of Model D exhibited superior performance, achieving an accuracy of 0.8670.

## 4. Discussion

This study examined the efficacy of action recognition of Taekwondo poomsae. This was achieved by examining various training and testing datasets. The performance of four models, namely Models A, B, C, and D, trained on 2D skeletal representations obtained by projecting 3D skeletons from diverse camera viewpoints, was evaluated and contrasted across distinct testing datasets. The evaluation outcomes of the 2D skeletal data obtained by projecting the 3D skeleton at 10° intervals across the viewpoints revealed that Model A achieved an accuracy of only 0.7997. In contrast, Models B, C, and D achieved accuracies of more than 0.96. This observation underscores the insufficiency of relying solely on frontal and lateral viewpoint data to recognize actions from other perspectives.

Next, the performance of the proposed model was evaluated using RGB images captured from the frontal and lateral viewpoints. Among the four models assessed, Model A again exhibited the lowest accuracies of 0.5795 and 0.6549 for the joint and bone heatmap models, respectively. In contrast, Model D demonstrated the highest accuracy of 0.8705 in the joint heatmap model, and Model C achieved the highest accuracy of 0.8761 in the bone heatmap model. This highlights the potential enhancement of the recognition performance achieved via the incorporation of projection data from different viewpoints. The assessment encompassed image data captured from random viewpoints, and 2D skeletal representations were extracted from images obtained using HRNet from those viewpoints. Model C achieved the highest joint heatmap precision of 0.9381, while Model D attained a peak skeletal heatmap precision of 0.8670. The observed decline in the accuracy of the image data evaluation can be attributed to the disparities between the 2D skeletal representations obtained via the projection of 3D skeletons and those obtained using the estimation algorithm. Despite efforts to align them by discarding significantly different keypoints, inherent misalignments still affected the accuracy. The alignment challenge can be addressed by utilizing a generative adversarial network (GAN) model trained with a dataset comprising 2D skeletons obtained through the projection of 3D skeletons and corresponding 2D skeletons extracted from the image.

To understand the limitations of Model A, we conducted qualitative analysis, which revealed that Model A struggles to accurately recognize actions under varying camera viewpoints. This limitation becomes particularly pronounced when assessing Taekwondo forms from non-frontal and non-profile angles. Model A’s shortcomings are evident as it frequently misinterprets complex movements, leading to lower accuracy scores. In contrast, Models C and D exhibit outstanding accuracy in action recognition. Qualitative analysis indicates that their robustness stems from their ability to generalize well across different camera perspectives. Even when faced with challenging viewpoints, they consistently recognize key actions. This qualitative insight underscores the potential of these models in real-world applications, where camera angles may not always be fixed. To qualitatively assess the viewpoint sensitivity of the proposed model, we analyzed their performance across a range of camera perspectives. While quantitative results indicate some sensitivity to different viewpoints, qualitative analysis reveals that the models may struggle to achieve complete accuracy when trained on limited data.

To compare the proposed approach and the existing models, the latter were trained on sample images acquired from two RGB-D cameras positioned frontally and laterally. The images were employed to extract 2D skeletal representations that were used for training. A comparative assessment was conducted by juxtaposing the models on a test dataset of random-view RGB images, where Model C exhibited better performance than the existing models by more than 10%. This observation underscores the high recognition performance of the proposed method for Taekwondo poomsae from arbitrary viewpoints. Thus, the motion-recognition methodology demonstrates high precision across distinct viewpoints and datasets. Additionally, the recognition performances of Models C and D were superior to those of other variants. Furthermore, compared with the previously reported models, the proposed model exhibited superiority in action recognition, which highlights the efficacy of the proposed approach in real-world scenarios.

This study employed various training and testing datasets, yet the variability in the data may not fully encapsulate the complexity and diversity of real-world scenarios. However, we believe that the proposed model holds potential feasibility in other domains as well. It is important to note that validating the proposed model in these alternative domains requires a significant allocation of resources. The evaluation of this study focused on Taekwondo poomsae movements, and the generalizability of the proposed approach to other action recognition tasks remains to be explored. The action dataset TUAD-3D, which was curated by Taekwondo experts, was constructed for the purpose of developing and validating the proposed methodology. However, the application of the proposed method to the assessment of Taekwondo poomsae performed by general individuals requires a diverse set of action samples. This is because individuals’ executions of the movements may exhibit significant variations. Collecting data from such individuals is a task that will be addressed in our future work.

Future research can benefit from integrating a wider array of data sources to capture diverse lighting conditions, backgrounds, and environmental factors. Given that the proposed action recognition algorithm relies on skeleton data, real-world environmental factors, such as lighting conditions and background noise, do not directly affect the recognition performance. Those environmental factors are removed in the process of skeleton extraction. However, it is important to note that these real-world environmental factors can still have an impact on the accuracy of 2D skeletons extracted through pose-estimation algorithms from images.

The computational cost of the proposed model was measured in FLOPs (floating-point operations per second). The model comprises two million parameters, reflecting a balanced model complexity. Both model training and inference were performed on a high-performance computing system equipped with an AMD^®^ Ryzen Threadripper 3960X 24-core processor, 48 CPUs, two GeForce RTX 3090 GPUs, and 96.0 GB of memory. The model’s average inference speed was measured at 0.125 s, demonstrating efficient real-time performance.

## 5. Conclusions

This study constructed a TUAD-3D dataset by employing full-body motion-capture suits to collect accurate 3D skeletal data. This dataset contained 7680 samples and included 16 fundamental techniques performed by 40 Taekwondo experts. The model effectively synthesized 2D skeletal representations from the collected 3D skeletal data and integrated multiple viewpoints during the training process. This approach ensured consistent and reliable model performance, regardless of the observer’s angles and positions. Through a comprehensive evaluation of various action recognition networks, we observed that two of the model variants, Models C and D, which were trained using 3D skeleton projection, exhibited higher accuracy. The assessment results demonstrated the superiority of the proposed model over those reported previously, highlighting its effectiveness in Taekwondo poomsae action recognition and classification. Furthermore, analysis across different viewpoints and datasets revealed the significance of optimal camera viewpoint selection for training, which influenced the model performance. However, a decline in accuracy was observed during the model evaluation of image data, owing to inherent disparities between the 2D skeletal representations obtained via 3D skeleton projections and those estimated by the algorithm. Despite efforts to align these skeletal representations, their accuracies remained affected. In conclusion, this study contributes to the advancement of action recognition technology in the context of Taekwondo poomsae. The proposed model demonstrated robust performance across various viewpoints and datasets, highlighting its potential for real-world applications. Considering factors such as viewpoint selection and data alignment, further investigation and refinement of action recognition models can enhance their accuracy and performance in the field of human motion analysis.

## Figures and Tables

**Table 1:** Viewpoint configuration of 2D skeletons of four training datasets.

ID	Viewpoint Configuration	Number of Viewpoints	Number of Training Data
Model A	0°, 90°	2	12,360
Model B	0°, 90°,180°,270°	4	24,720
Model C	0°, 45°, 90°, 135°,⋯, 315°	8	49,440
Model D	0°, 10°, 20°, 30°, ⋯, 350°	36	222,480

**Table 2:** Performance evaluation results of the joint heatmap model tested using a random-projection 2D skeletal dataset.

Model	*Precision*	*Recall*	*F1-Score*	*Accuracy*
Model A	0.8611	0.8398	0.8373	0.7997
Model B	0.9680	0.9669	0.9670	0.9669
Model C	0.9769	0.9764	0.9765	0.9764
Model D	0.9803	0.9802	0.9802	0.9802

**Table 3:** Performance evaluation results of the bone heatmap model tested using a random-projection 2D skeletal dataset.

Model	*Precision*	*Recall*	*F1-Score*	*Accuracy*
Model A	0.8611	0.8398	0.8373	0.7997
Model B	0.9686	0.9682	0.9682	0.9682
Model C	0.9769	0.9764	0.9765	0.9764
Model D	0.9786	0.9783	0.9784	0.9783

**Table 4:** Performance evaluation results of the joint heatmap model using the 2D skeletal dataset extracted from RGB images.

Model	*Precision*	*Recall*	*F1-Score*	*Accuracy*
Model A	0.7638	0.7638	0.5854	0.5795
Model B	0.8761	0.8533	0.8500	0.8516
Model C	0.8705	0.7647	0.7763	0.7626
Model D	0.8998	0.8717	0.8706	0.8705

**Table 5:** Performance evaluation results of bone heatmap model using the 2D skeletal dataset extracted from RGB images.

Model	*Precision*	*Recall*	*F1-Score*	*Accuracy*
Model A	0.7453	0.6559	0.6549	0.6549
Model B	0.8750	0.8303	0.8300	0.8294
Model C	0.8903	0.8766	0.8752	0.8761
Model D	0.8891	0.8743	0.8717	0.8732

**Table 6:** Performance evaluation results of the joint heatmap model using the 2D skeletal dataset extracted from the RGB images captured from random viewpoints.

Model	*Precision*	*Recall*	*F1-Score*	*Accuracy*
Model A	0.8298	0.8185	0.7944	0.8398
Model B	0.9217	0.9009	0.9037	0.9010
Model C	0.9432	0.9381	0.9384	0.9381
Model D	0.8977	0.8702	0.8682	0.8623

**Table 7:** Performance evaluation results of the bone heatmap model using the 2D skeletal dataset extracted from the RGB images captured from random viewpoints.

Model	*Precision*	*Recall*	*F1-Score*	*Accuracy*
Model A	0.7142	0.6222	0.6095	0.6041
Model B	0.8665	0.7862	0.7930	0.7715
Model C	0.8848	0.8471	0.8482	0.8419
Model D	0.9040	0.8736	0.8764	0.8670

**Table 8:** Performance comparison among the proposed and previous models using synthetic two-dimensional skeletal test dataset.

Model	*Precision*	*Recall*	*F1-Score*	*Accuracy*
stgcn [29]	0.6926	0.5911	0.5957	0.5911
stgcn++ [33]	0.6700	0.5327	0.5516	0.5327
ctrgcn [26]	0.7129	0.5805	0.6060	0.5805
aagcn [34]	0.7417	0.6471	0.6571	0.6471
posec3d [22]	0.7453	0.5818	0.6118	0.5818
Proposed (Model C)	0.9769	0.9764	0.9765	0.9764
Proposed (Model D)	0.9786	0.9783	0.9784	0.9783

**Table 9:** Performance comparison among the proposed and previous models using random-viewpoint RGB image test dataset.

Model	*Precision*	*Recall*	*F1-Score*	*Accuracy*
stgcn [29]	0.7257	0.6915	0.6715	0.6667
stgcn++ [33]	0.8144	0.7210	0.7417	0.7058
ctrgcn [26]	0.7944	06509	0.6587	0.6275
aagcn [34]	0.8040	0.7442	0.7541	0.7261
posec3d [22]	0.8825	0.7520	0.7865	0.7340
Proposed (Model C)	0.8848	0.8471	0.8482	0.8419
Proposed (Model D)	0.9040	0.8736	0.8764	0.8670

## Data Availability

Data cannot be provided, owing to data security reasons.
